# Supplementary material for: Study design and the sampling of deleterious rare variants in biobank-scale datasets
Source: bioRxiv. 2025 Jan 29:2024.12.02.626424. Originally published 2024 Dec 2. Preprint. [Version 2] doi: 10.1101/2024.12.02.626424 (PMC11642817; doi:10.1101/2024.12.02.626424)
Supplement: Supplement 1 [file NIHPP2024.12.02.626424v2-supplement-1.pdf]

bioRxiv preprint doi: https://doi.org/10.1101/2024.12.02.626424; this version posted January 29, 2025. The copyright holder for this preprint (which was not certified by peer review) is the author/funder, who has granted bioRxiv a license to display the preprint in perpetuity. It is made available under aCC-BY 4.0 International license.

# 1 Supplemental Information

## 1.1 Extended theoretical methods

Here, we provide a detailed description of our theoretical methods. We model the movement, reproduction, and death of the carriers of a rare deleterious allele. These carriers are generated by mutations in a much larger population of wild-type individuals. By explicitly modeling only the rare carriers, we can approximate the evolution of their spatial distribution as a *superprocess*. We use the recent results of Friesen (2023) to find the equilibrium moment generating functional of the spatial distribution of deleterious alleles at mutation-selection-migration-drift balance.Then, we apply a model of spatial sampling to compute the expected site frequency spectra for varying spatial sampling schemes.

In the following, we will assume that all parameters (i.e., population density, mutation rate, selection coefficient, and dispersal diffusion coefficient) are spatially and temporally homogeneous. It is straightforward to specify a more general model and to apply the same procedure we outline below to calculate its site frequency spectra. However, the numerical calculations become significantly more complicated. Therefore, we focus on getting intuition from the scaling results in the homogeneous case and leave generalizations to future work.

### 1.1.1 Population genetic model

We consider a population of organisms living in a habitat $H$. Here we will focus on 2−dimensional continuous habitats, so that $H \subset \mathbb{R}^2$, but most of the theory applies to more general habitats. For simplicity, in our numerical calculations we will use a toroidal habitat of length $L$, i.e., $H = [0, L]^2$ with periodic boundary conditions. Let $\rho_N$ be the population density measure so that the number of individuals living in a region $A \subset H$ is given by $N(A) = \int_A \rho_N(\vec{x})dx$. The total population size is $N \equiv \int_H \rho_N(\vec{x})dx$. We assume that $\rho_N$ is large and stably maintained by ecological forces so that we can neglect random fluctuations in population density due to migration, births, and deaths.

We are interested in tracking the number and spatial distribution of carriers of a rare deleterious variant with fitness cost $s > 0$. We will restrict ourselves to the weak-selection regime, $s \ll 1$. With this assumption, carriers of the deleterious allele have $1 - s$ offspring on average, compared to 1 for non-carriers. By focusing on rare alleles, we can neglect dominance effects because homozygous carriers should make up a negligible fraction of the carriers. We assume that wild-type alleles undergo mutation to the deleterious allele at rate $\mu$ per generation per individual. Thus, we model the influx of *de novo* mutations as a Poisson point process with intensity $\mu\rho_N(\vec{x})$. This approximation neglects the reduction in the mutation supply due to the fact that some fraction of the $\rho_N$ might already carry the mutation. However, if $\mu \ll s$, the effect of selection dominates the resulting reduction in mutation supply and we can neglect it. We will similarly neglect back-mutation from the mutant to the wild-type.

For mathematical tractability, we will assume that carriers of the deleterious allele reproduce, die, and move about the habitat independent of one another and of the background of wild-type individuals. This assumption is justified as long as the deleterious allele remains rare. Given the rare-allele assumption, we model the movement of an individual carrier

bioRxiv preprint doi: https://doi.org/10.1101/2024.12.02.626424; this version posted January 29, 2025. The copyright holder for this preprint (which was not certified by peer review) is the author/funder, who has granted bioRxiv a license to display the preprint in perpetuity. It is made available under aCC-BY 4.0 International license.

as a continuous-time Markov process on $H$ with infinitessimal generator $\sigma^2\nabla^2$ (i.e., translation invariant, isotropic diffusion), independent of the positions of other carriers. This simplification is similar to Haldane's branching process approximation to the Wright-Fisher process (Haldane, 1927), and has since been applied in spatial population genetic modeling (for instance, Novembre and Slatkin, 2009).

In a continuous habitat we need to choose a suitable definition of 'rare' that accounts for spatial variation in the mutant frequency. To wit, let $\sigma$ be the average distance an individual will move in one generation (more formally, the root mean squared difference between parent and offspring birth locations). We assume that the number of carriers in a ball $B_\sigma(\vec{x})$ with radius $\sigma$ about any point $\vec{x} \in H$ is small compared to $N(B_\sigma(\vec{x}))$, Wright's neighborhood size (Wright, 1946). As $\sigma$ is the shortest length scale in the model, it is reasonable to assume that fluctuations in allele frequency at distances shorter than this scale will be short-lived and not contribute to the long-term evolution of the population.

Having defined the mutational process by which carriers are generated and the migration process by which they move around the habitat, it remains to define the mechanism by which they die and reproduce. Consistent with our assumption that carriers behave independently, we will model reproduction as a continuous-state branching process. Combining this with our Markov process for movement yields a superprocess model of the evolution of the spatial distribution of carriers (Watanabe, 1968; Dawson, 1993). For overviews of superprocesses and their properties, see Le Gall (1999); Etheridge (2000). If we add an influx of particles according to the mutational point process with intensity $\mu\rho_N$, we have a superprocess with immigration (Kawazu and Watanabe, 1971). (Confusingly, in the superprocess literature, our mutation process is called "immigration" and the migration process is sometimes called "mutation".) We now define this process and then give the main result (Eqs. 10–12) needed to calculate sample properties.

A superprocess $\{Z_t\}$ is a measure-valued random process. That is, $\{Z_t\}$ is a set of measures on the habitat $H$ indexed by time, $t$. Measures are defined by how they integrate functions over their domain. Accordingly, we introduce the inner product $\langle Z, f\rangle \in \mathbb{R}$, defined as:

$$\langle Z, f\rangle = \int_H f(\vec{x})dZ(\vec{x}), \tag{7}$$

where $Z$ is a finite measure on $H$, and $f : H \to \mathbb{R}$ is a measurable function on $H$ (Le Gall, 1999). The probability distribution of a superprocess at time $t$ is characterized by its *moment generating functional* (MGF), $\Phi_t$, defined as:

$$\Phi_t[f] = \mathbb{E}\left[\exp\left(\langle Z_t, f\rangle\right)\right]. \tag{8}$$

Just as the derivatives of the moment generating function of a random variable gives its moments, the functional derivatives of $\Phi_t$ with respect to $f$ give moments of the inner product $\langle Z_t, f\rangle$.

In our model, $Z_t$ measures the number of carriers in a region of space. For a region $A \subseteq H$, we define an indicator function $I_A(\vec{x}) = 1$ for $\vec{x} \in A$ and zero otherwise, so that:

$$\langle Z_t, I_A\rangle = \#\text{ of carriers in } A, \text{ and } \langle Z_t, I_H\rangle = \text{total } \#\text{ of carriers}. \tag{9}$$

By analogy, $\langle Z_t, f\rangle$, for an arbitrary non-negative $f$, gives the counts of carriers according to their positions by the weighting function $f$.

bioRxiv preprint doi: https://doi.org/10.1101/2024.12.02.626424; this version posted January 29, 2025. The copyright holder for this preprint (which was not certified by peer review) is the author/funder, who has granted bioRxiv a license to display the preprint in perpetuity. It is made available under aCC-BY 4.0 International license.

Friesen (2023) has recently shown that subcritical superprocesses (i.e., ones where the measure decays exponentially) with immigration tend to a stationary distribution, subject to various technical conditions. In particular, for our process with mutational supply intensity $\mu\rho_N$, diffusion coefficient $\sigma^2$, and selection coefficient $s$,

$$\lim_{t\to\infty} \Phi_t[f] = \Phi[f] \equiv \exp\left(\int_0^\infty \langle \mu\rho_N, u_t\rangle \, dt\right), \tag{10}$$

where $u_t$ is the solution to the semilinear PDE:

$$\frac{\partial}{\partial t} u_t(\vec{x}) = \sigma^2 \nabla^2 u - su + u^2, \tag{11}$$

subject to initial condition:

$$u_0(\vec{x}) = f(\vec{x}). \tag{12}$$

The function $u$ does not have a direct biological interpretation, though its associated PDE incorporates several features of the evolutionary process (including selection and drift).

The stationary MGF, $\Phi$, completely characterizes the counts and spatial distribution of carriers of the deleterious alleles of a population at steady-state. These patterns are due to the balance between the forces of mutation, selection, genetic drift, and migration. In the rest of this section, we will show how Eq. 10 can be used to calculate the expected site frequency spectrum for a spatially localized sample from the population.

#### 1.1.2 Spatial sampling the allele frequency distribution

We now connect the superprocess model of allele frequencies to the site frequency spectrum of a finite sample. We are interested in geographically biased samples, where the probability that an individual is sampled depends on its location according to a *sampling density*. Let the kernel function $g(\cdot)$ be the shape of the sampling density so that $\int_H g(x)dx = 1$. To capture the effect of broad versus narrow sampling, we will consider sampling kernels with a scale parameter $w$, which represents the typical distance between sampled individuals. In particular, for sampling on a torus, we will use a bivariate wrapped Gaussian sampling kernel with standard deviation $w$.

Note that the sampling density is a measure of the *a priori* sampling effort across space, rather than the realized locations of sampled individuals. In the following, we assume that we do not have access to the locations of our sampled individuals. However, this framework could be extended to consider the joint statistics of samples taken from multiple known locations.

We define the expected value of the $k$'th element of the site frequency spectrum (SFS) as the expected fraction of sites with $k$ copies of the deleterious allele ($\xi_k^{(n)} = E[Pr(K = k)]$). Assuming that the samples are taken independently from the population with replacement, the number of copies of the deleterious allele is the sum of $n$ Bernoulli trials with a random probability of success:

$$\xi_k^{(n)} = \mathbb{E}\left[\binom{n}{k} P^k (1-P)^{n-k}\right], \tag{13}$$

bioRxiv preprint doi: https://doi.org/10.1101/2024.12.02.626424; this version posted January 29, 2025. The copyright holder for this preprint (which was not certified by peer review) is the author/funder, who has granted bioRxiv a license to display the preprint in perpetuity. It is made available under aCC-BY 4.0 International license.

where $P$ is a random variable representing the probability any particular sampled allele is deleterious.

The distribution of $P$ depends on (1) the locations of carrier individuals characterized by the superprocess $Z$, and (2) the probability that we sample a carrier given its location. For a sample taken uniformly from a region $A \subseteq H$, $P$ is the fraction of carriers in $A$:

$$P(A) = \frac{\text{\# of carriers in } A}{\text{total \# of individuals in } A} = \frac{\langle Z, I_A \rangle}{N(A)}. \tag{14}$$

If, instead, the sample is taken by choosing among a countable set of regions $\{A_i\}$ according to probabilities $\{g_i\}$ and then sampling uniformly within the chosen region,

$$P = \sum_i g_i \frac{\langle Z, I_{A_i} \rangle}{N(A_i)} = \left\langle Z, \sum_i \frac{g_i I_{A_i}}{N(A_i)} \right\rangle, \tag{15}$$

where the second equality uses the linearity of inner products. Taking the limit that sampling probabilities vary continuously according to a localized sampling density, we have

$$P \to \left\langle Z, \rho_N^{-1} g(\cdot) \right\rangle, \tag{16}$$

so that the sampling probability is an inner product of the random measure $Z$ with the population-scaled sampling density.

Therefore, using Eqs. 8, 10, and 16, the moment generating function of $P$ at steady-state is given by

$$\begin{aligned} \mathrm{MGF}_P(z) &\equiv \mathbb{E}\left[\exp\left(zP\right)\right] \\ &= \lim_{t\to\infty} \mathbb{E}\left[\exp\left(\left\langle Z_t, \rho_N^{-1} g(\cdot)\right\rangle\right)\right] \\ &= \Phi\left[\left(\frac{z}{\rho_N}\right) g(\cdot)\right] \qquad (17) \\ &= \exp\left(\int_0^\infty \langle \mu\rho_N, u_t\rangle \, dt\right), \qquad (18) \\ &= \exp\left(\mu\rho_N \int_0^\infty \int_H u(\vec{x}, t) d^2\vec{x} dt\right), \qquad (19) \end{aligned}$$

where $u_t$ solves Eq. 11 with initial condition

$$u_0(\vec{x}) = \left(\frac{z}{\rho_N}\right) g(\vec{x}). \tag{20}$$

In the following sections, we will use these equations to solve for key properties of the distribution of $P$ in order to evaluate the SFS per Eq. 13.

### 1.1.3 Moments of the allele frequency distribution

Here, we aim to calculate the log of the MGF of $P$ by way of Eq. 19. We consider a habitat $H = [-L/2, L/2]^2$ (for $L \in \mathbb{R}$) with periodic boundary conditions. For mathematical

bioRxiv preprint doi: https://doi.org/10.1101/2024.12.02.626424; this version posted January 29, 2025. The copyright holder for this preprint (which was not certified by peer review) is the author/funder, who has granted bioRxiv a license to display the preprint in perpetuity. It is made available under aCC-BY 4.0 International license.

convenience, we will work in Fourier space. First, we take a Fourier transform of $u(\vec{x}, t)$ over both time and space:

$$\hat{u}(\vec{\nu}, \omega) \equiv \int_0^\infty \int_H u(\vec{x}, t) \exp\left(-2\pi i \left(\frac{\vec{\nu} \cdot \vec{x}}{L} + \omega t\right)\right) \frac{d^2\vec{x}}{L^2} dt \tag{21}$$

where $\omega \in \mathbb{R}$ (temporal frequency) and $\vec{\nu} \in \mathbb{Z}^2$ (spatial frequency). Applying the same transformation to the PDE in Eq. 11 gives:

$$\left(2\pi i\omega + \left(\frac{2\pi\sigma}{L}\vec{\nu}\right)^2 + s\right)\hat{u} = \hat{u} * \hat{u} + \frac{z}{\rho_N}\hat{g}(\vec{\nu}). \tag{22}$$

It then follows from Eqs. 19 and 21 that:

$$\log \mathrm{MGF}_P(z) = \mu\rho_N L^2 \hat{u}(\vec{0}, 0), \tag{23}$$

and as such we aim to solve for the value of $\hat{u}$ at the origin.

To proceed, we calculate a perturbative expansion of $\hat{u}$ in powers of $z$, up to the second order:

$$\hat{u} = z\hat{u}_1 + z^2\hat{u}_2 + \mathcal{O}(z^3). \tag{24}$$

Substituting into Eq. 22 gives:

$$\hat{u}_1 = \left(2\pi i\omega + \left(\frac{2\pi\sigma}{L}\vec{\nu}\right)^2 + s\right)^{-1} \frac{1}{\rho_N}\hat{g}(\vec{\nu}) \tag{25}$$

and

$$\hat{u}_2 = \left(2\pi i\omega + \left(\frac{2\pi\sigma}{L}\vec{\nu}\right)^2 + s\right)^{-1} \left(\hat{u}_1 * \hat{u}_1\right). \tag{26}$$

Evaluating at the origin, we have:

$$\hat{u}_1(\vec{0}, 0) = \frac{\hat{g}(\vec{0})}{s\rho_N} = \frac{1}{s\rho_N L^2} \tag{27}$$

and

$$\hat{u}_2(\vec{0}, 0) = s^{-1}\left(\hat{u}_1 * \hat{u}_1\right)(\vec{0}, 0) \tag{28}$$

$$= s^{-1}\sum_{\vec{\nu}} \left(\int_{-\infty}^{\infty} \frac{1}{(2\pi\omega)^2 + \left(\left(\frac{2\pi\sigma}{L}\vec{\nu}\right)^2 + s\right)^2} d\omega\right) \frac{\hat{g}(\vec{\nu})\hat{g}(-\vec{\nu})}{{\rho_N}^2} \tag{29}$$

$$= \frac{1}{2s^2{\rho_N}^2} \sum_{\vec{\nu}\in\mathbb{Z}^2} \frac{\hat{g}(\vec{\nu})\hat{g}(-\vec{\nu})}{\left(\frac{2\pi\ell_c}{L}\vec{\nu}\right)^2 + 1}, \tag{30}$$

where the last line introduces the critical distance $\ell_c = \sqrt{\frac{\sigma^2}{s}}$. We refer to this value as the *characteristic length scale* (see section 3.1 in the main text).

bioRxiv preprint doi: https://doi.org/10.1101/2024.12.02.626424; this version posted January 29, 2025. The copyright holder for this preprint (which was not certified by peer review) is the author/funder, who has granted bioRxiv a license to display the preprint in perpetuity. It is made available under aCC-BY 4.0 International license.

From here, we can calculate the mean and variance of $P$. We find that the mean allele frequency is independent of the sampling scheme:

$$\mathbb{E}[P] = \frac{d}{dz} MGF_P\big|_{z=0} \tag{31}$$
$$= \mu \rho_N L^2 \hat{u}_1(\vec{0}, 0) \tag{32}$$
$$= \frac{\mu}{s}. \tag{33}$$

On the other hand, the variance is given by:

$$\mathbb{V}[P] = 2\mu\rho_N L^2 \hat{u}_2(\vec{0}, 0) \tag{34}$$
$$= \frac{\mu}{s^2 \rho_N L^2} \sum_{\vec{\nu} \in \mathbb{Z}^2} \frac{L^4 \hat{g}(\vec{\nu})\hat{g}(-\vec{\nu})}{\left(\frac{2\pi \ell_c}{L}\vec{\nu}\right)^2 + 1}, \tag{35}$$

and so is dependent on the sampling kernel $g(\cdot)$. If sampling is uniform over the habitat, $\hat{g}(\vec{\nu}) = L^{-2}\delta_{\vec{\nu}}$. Thus,

$$\mathbb{V}[P] = \frac{\mu}{s^2 \rho_N L^2} = \frac{\mu}{s^2 N}. \tag{36}$$

On the other hand, if sampling follows a wrapped normal distribution with scale $w$,

$$\mathbb{V}[P] = \frac{\mu}{s^2 \rho_N L^2} \sum_{\vec{\nu} \in \mathbb{Z}^2} \frac{\exp\left(-\left(\frac{2\pi w}{L}\vec{\nu}\right)^2\right)}{\left(\frac{2\pi \ell_c}{L}\vec{\nu}\right)^2 + 1}. \tag{37}$$

For $w \gtrsim L$, the numerator of the sum falls off rapidly with $|\vec{\nu}|$ and we converge to the uniform sampling result. For $\ell_c \gg L$, the denominator grows large for $|\vec{\nu}| > 0$ and we again converge to the uniform sampling result. For $w, \ell_c \ll L$, we can approximate the sum with an integral:

$$\mathbb{V}[P] \approx \frac{\mu}{s^2 \rho_N} \int_{\mathbb{R}^2} \frac{\exp\left(-\left(\frac{2\pi w}{L}\vec{\nu}\right)^2\right)}{\left(\frac{2\pi \ell_c}{L}\vec{\nu}\right)^2 + 1} \frac{d^2\vec{\nu}}{L^2} \tag{38}$$
$$= \frac{\mu}{s^2 \rho_N 4\pi \ell_c^2} \exp\left((w/\ell_c)^2\right) E_1\left((w/\ell_c)^2\right). \tag{39}$$

For $w \gg l_c$, this is approximately $\mu/(s^2 \rho_N 4\pi w^2)$, which implies that we converge to the uniform sampling result when $w \approx L/(2\sqrt{\pi}) \approx 0.3L$. For $w \to 0$, the expression diverges as we integrate over very large frequencies, which correspond to very small length scales where our model breaks down. We can remedy this by imposing a cutoff on $|\vec{\nu}|$. The smallest length scale our model can sensibly talk about is $\sigma$, the per-generation dispersal distance. Thus, a reasonable cutoff is $|\vec{\nu}| = L/\sigma$.

We validate these expressions by comparing results for $\mathbb{E}[P]$ and $\mathbb{E}[P^2]$ in simulations to their respective values under the model, and find a close correspondence (Fig. S4). Additionally, as the sampling width approaches the habitat width in the simulations, values of $\mathbb{E}[P^2]$ approach expected values under uniform sampling, as expected.

bioRxiv preprint doi: https://doi.org/10.1101/2024.12.02.626424; this version posted January 29, 2025. The copyright holder for this preprint (which was not certified by peer review) is the author/funder, who has granted bioRxiv a license to display the preprint in perpetuity. It is made available under aCC-BY 4.0 International license.

684 ### 1.1.4 Effective parameters and the sample SFS

685 Having calculated the first two moments of $P$, we will now use them to approximate the full
686 distribution of $P$ and use this to calculate the SFS of a sample of finite size. We will assume
687 that $P$ approximately follows a Gamma distribution whose parameters we can calculate
688 from the first two moments. If sampling is uniform over the habitat, it can be shown that
689 this holds exactly (see section 1.1.6), and we show via simulation that this assumption is
690 reasonable for non-uniform sampling as well (Figs. S2 and S3). Thus, we assume $P$ follows:

$$P \sim \text{Gamma}(\theta_E, \gamma_E), \tag{40}$$

691 and we will refer to the shape and rate parameters, $\theta_E$ and $\gamma_E$, as the *effective mutation*
692 *supply* and *effective selection intensity*, respectively. The motivation behind these names
693 will be clarified by their derivation.

694 We derive the form of these parameters for both the uniform sampling and wrapped
695 normal sampling cases using the method of moments applied to previous results (Eqs. 33-
696 39). When sampling is spatially uniform, we have:

$$\theta_E = \frac{\mu^2/s^2}{\mu/(s^2 N)} = \mu N, \tag{41}$$

697 and

$$\gamma_E = \frac{\mu/s}{\mu/(s^2 N)} = sN. \tag{42}$$

698 When sampling follows a wrapped normal distribution with scale $w$, we have:

$$\begin{aligned}\theta_E &= \frac{\mu^2/s^2}{(\mu/(s^2\rho_N 4\pi\ell_c^2))\exp\left((w/\ell_c)^2\right) E_1\left((w/\ell_c)^2\right)} \\ &= \mu\frac{4\pi\rho_N\ell_c^2}{\exp((w/\ell_c)^2)E_1((w/\ell_c)^2)} \qquad (43)\\ &= \mu\rho_N\ell_c^2\lambda, \qquad (44)\end{aligned}$$

699 where:

$$\lambda \equiv \frac{4\pi}{\exp((w/\ell_c)^2)E_1((w/\ell_c)^2)}. \tag{45}$$

700 Similarly, we have:

$$\begin{aligned}\gamma_E &= \frac{\mu/s}{(\mu/(s^2\rho_N 4\pi\ell_c^2))\exp\left((w/\ell_c)^2\right) E_1\left((w/\ell_c)^2\right)} \\ &= s\frac{4\pi\rho_N\ell_c^2}{\exp((w/\ell_c)^2)E_1((w/\ell_c)^2)} \qquad (46)\\ &= s\rho_N\ell_c^2\lambda. \qquad (47)\end{aligned}$$

701 The compound scale factor $\lambda$ (which we refer to as the *sampling effect scalar*) captures
702 all spatial sampling aspects of the problem.

bioRxiv preprint doi: https://doi.org/10.1101/2024.12.02.626424; this version posted January 29, 2025. The copyright holder for this preprint (which was not certified by peer review) is the author/funder, who has granted bioRxiv a license to display the preprint in perpetuity. It is made available under aCC-BY 4.0 International license.

To summarize, we define the distribution of $P$ for the uniform sampling case as:

$$P \sim \text{Gamma}(\mu N, sN), \tag{48}$$

and for the wrapped Normal case as:

$$P \sim \text{Gamma}(\mu \rho_N \ell_c^2 \lambda, s\rho_N \ell_c^2 \lambda). \tag{49}$$

For a sample of $n$ haploid genomes, let $K \in \{0, \ldots, n\}$ be a random variable representing the number of sampled copies of the deleterious allele in the focal site. Recall from Eq. 13 that the number of copies of the deleterious allele is the sum of $n$ Bernoulli trials with probability of success $P$, or equivalently, $K \sim \text{Binom}(n, P)$. For large $n$ and small $P$, this is approximately $K \sim \text{Pois}(nP)$. Then, from properties of Gamma-Poisson mixtures, allele counts in a finite sample of size $n$ follow:

$$K \sim \text{NegBin}\left(\mu N, \frac{sN}{sN + n}\right), \tag{50}$$

for the uniform sampling case, and:

$$K \sim \text{NegBin}\left(\mu \rho_N \ell_c^2 \lambda, \frac{s\rho_N \ell_c^2 \lambda}{s\rho_N \ell_c^2 \lambda + n}\right), \tag{51}$$

for the wrapped Normal sampling case. We can use these distribution to calculate elements of the SFS as:

$$\xi_k^{(n)} \equiv \Pr\{K = k\}. \tag{52}$$

The remainder of the results follow from these expressions.

### 1.1.5 Derivation of summary statistics

Having derived the form of the SFS, we can now obtain expressions for various population genetic summary statistics. We will show explicit derivations only in the case of wrapped Normal sampling, for brevity, though similar derivations can be obtained easily for the uniform sampling case. First, we consider the expected proportion of variant sites in a sample, or equivalently the probability that a particular allele segregates in a sample of size $n$. This follows from Eq. 51:

$$\begin{aligned} \Pr\{K > 0\} &= 1 - \Pr\{K = 0\} \\ &= 1 - \left(\frac{\gamma_E}{\gamma_E + n}\right)^{\theta_E} \end{aligned} \tag{53}$$

Mean allele frequency is invariant to the scale of sampling:

$$\begin{aligned} \mathbb{E}\left[\frac{K}{n}\right] &= \frac{1}{n} \cdot \theta_E \cdot \frac{n}{\gamma_E + n} \cdot \frac{\gamma_E + n}{\gamma_E} \\ &= \frac{\theta_E}{\gamma_E} \\ &= \frac{\mu}{s}, \end{aligned} \tag{54}$$

bioRxiv preprint doi: https://doi.org/10.1101/2024.12.02.626424; this version posted January 29, 2025. The copyright holder for this preprint (which was not certified by peer review) is the author/funder, who has granted bioRxiv a license to display the preprint in perpetuity. It is made available under aCC-BY 4.0 International license.

though the mean frequency of *non-monomorphic* alleles does vary according to the sampling design. We obtain an expression for the conditional mean as follows:

$$\begin{aligned}\mathbb{E}\left[\frac{K}{n}\middle|K>0\right] &= \frac{1}{n}\sum_k k\frac{\Pr(\{K=k\}\cap\{K>0\})}{\Pr\{K>0\}}\\ &= \frac{\mathbb{E}[K/n]}{\Pr\{K>0\}}\\ &= \frac{\mu/s}{1-\left(\frac{\gamma_E}{\gamma_E+n}\right)^{\theta_E}}. \end{aligned} \tag{55}$$

Heterozygosity is defined as the probability that two alleles are different from one another, and so it follows from the distribution of $P$ rather than the SFS. Accordingly, we calculate expected heterozygosity as follows, using Eq. 40:

$$\mathbb{E}[2P(1-P)] = 2\mathbb{E}[P] - 2\left[\mathbb{V}[P] + \mathbb{E}[P]^2\right] \tag{56}$$

$$= \frac{2\theta_E}{\gamma_E}\left[1-\left(\frac{1-\theta_E}{\gamma_E}\right)\right]. \tag{57}$$

We calculate cumulative MAF, which is informative of burden test power, following the definition of Wang et al. (2014). In the context of our theoretical results, this has the form:

$$\text{MAF}_{cumulative} = 1 - \prod_i^{2N}\left(1-\frac{i}{n}\right)^{L\cdot\xi_i}, \tag{58}$$

where $\xi_i$ is the expected number of variants at count $i$ per basepair and $L$ is the length of the genomic region (bp).

We note that this expression for cumulative MAF is approximately proportional to the expected per-site allele frequency and heterozygosity. To see this, we first express the cumulative MAF in terms of $\mathbb{E}[P]$, for small $i/n$ and small $L\mathbb{E}[P]$:

$$\begin{aligned}1-\prod_i^n\left(1-\frac{i}{n}\right)^{L\cdot\xi_i} &= 1-\exp\left(\ln\left(\prod_i^n\left(1-\frac{i}{n}\right)^{L\cdot\xi_i}\right)\right)\\ &= 1-\exp\left(\sum_i^n \ln\left(1-\frac{i}{n}\right)^{L\cdot\xi_i}\right)\\ &= 1-\exp\left(L\sum_i^n \xi_i \ln\left(1-\frac{i}{n}\right)\right)\\ &\approx 1-\exp\left(-L\sum_i^n \xi_i\frac{i}{n}\right)\\ &\approx 1-\exp(-L\mathbb{E}[P])\\ &\approx 1-(1-L\mathbb{E}[P])\\ &\approx L\mathbb{E}[P].\end{aligned}$$

bioRxiv preprint doi: https://doi.org/10.1101/2024.12.02.626424; this version posted January 29, 2025. The copyright holder for this preprint (which was not certified by peer review) is the author/funder, who has granted bioRxiv a license to display the preprint in perpetuity. It is made available under aCC-BY 4.0 International license.

For small $\mathbb{E}[P]$, we have an expected heterozygosity of approximately $2\mathbb{E}[P]$. This implies that the cumulative MAF is approximately proportional to expected heterozygosity, with cumulative MAF being larger by a factor of $L/2$.

#### 1.1.6 Exact solution for the uniform sampling case

Here, we provide an exact derivation of the distribution of $P$ when sampling is uniform, providing motivation for our approximation that $P$ is approximately Gamma-distributed more generally. Eq. 11 is a nonlinear parabolic PDE and can not be solved in closed form for general initial conditions. However, if we sample uniformly over the habitat, $u_0(x)$ becomes constant and $\nabla^2 u = 0$, yielding a Bernoulli ODE for $u$, which we can solve exactly. Note that in our model, uniform sampling is equivalent to sampling from a panmictic population. This is because we are focused on rare alleles, which are assumed not to interact. For common alleles, local fixation changes the dynamics qualitatively and breaks this equivalence.

For uniform sampling, $g(x) = 1/L^2$, and the spatial derivative term vanishes so that Eqns. 11, 19, and 20 become:

$$\frac{d}{dt}u = -su + u^2 \tag{59}$$

$$\mathrm{MGF}_P(z) = \exp\left(\mu N \int_0^\infty u(t)dt\right) \tag{60}$$

$$u(0) = \frac{z}{\rho_N L^2} = \frac{z}{N} \tag{61}$$

At this point, we could solve Eq. 59 directly. Instead, we will motivate our approach to the non-uniform sampling case by finding a power series solution for $u$: $u(t) = \sum_{k=0}^{\infty} z^k u_k(t)$. Substituting into Eq. 59 and organizing the terms by powers of $z$, we can generate an infinite sequence of ODEs for the terms $\{u_k\}$. Starting with $k = 0$, we have

$$\frac{d}{dt}u_0 + su_0 = u_0^2, \tag{62}$$

with initial condition $u_0(0) = 0$. This has the trivial solution $u_0 = 0$.

For $k > 0$, we have

$$\frac{d}{dt}u_k + su_k = \sum_{\ell=1}^{k-1} u_\ell u_{k-\ell} \tag{63}$$

$$u_k(0) = \begin{cases} \frac{1}{N}, & k = 1 \\ 0, & \text{otherwise.} \end{cases} \tag{64}$$

Each equation in the hierarchy is a first-order linear ODE with a forcing term that depends only on the solutions to lower-order terms.

Then it can be shown by induction that for all $k \geq 1$, the following holds:

$$u_k = N^{-k}\left(\frac{1 - e^{-st}}{s}\right)^{k-1} e^{-st}. \tag{65}$$

bioRxiv preprint doi: https://doi.org/10.1101/2024.12.02.626424; this version posted January 29, 2025. The copyright holder for this preprint (which was not certified by peer review) is the author/funder, who has granted bioRxiv a license to display the preprint in perpetuity. It is made available under aCC-BY 4.0 International license.

Then, by property of a geometric series:

$$u(t) = \sum_{k=0}^{\infty} z^k u_k = \frac{\frac{z}{N} e^{-st}}{1 - \frac{z}{sN}(1 - e^{-st})}. \tag{66}$$

Substituting into equation Eq. 61 gives:

$$\log \mathrm{MGF}_P(z) = \mu N \int_0^{\infty} \frac{\frac{z}{N} e^{-st}}{1 - \frac{z}{sN}(1 - e^{-st})} dt \tag{67}$$

$$= -\mu N \log \left(1 - \frac{z}{sN}\right), \tag{68}$$

and so:

$$\mathrm{MGF}_P(z) = \left(1 - \frac{z}{sN}\right)^{-\mu N}. \tag{69}$$

Thus, $P$ follows a Gamma distribution with rate $sN$ and shape $\mu N$, which is consistent with predictions from classical population genetic models (Wright, 1940; Kimura and Ohta, 1978). Moreover, this foreshadows our results that the form of the SFS is determined by two compound parameters representing selection and mutation, respectively.

## 1.2 Extended simulation methods

Here, we provide details on our simulation methods. All simulation code and associated scripts are available at: https://github.com/NovembreLab/spatial_rare_alleles.

### 1.2.1 Spatial branching process simulations

Our first set of simulations is based on a branching process framework and aligns closely with our theoretical model. The habitat is a square of length $L$ with periodic boundary conditions. Consistent with our theoretical model, carriers appear *de novo* with rate $\mu \cdot \rho_N$, give birth with rate $1 - s$, and die at rate 1. Between events, dispersal of individuals occurs according to a Gaussian distribution with variance $\sigma^2 t$ where $t$ is the time between events. We sample alleles at random times at rate $r$, according to a wrapped Gaussian sampling kernel with scale parameter $w$. We implement these simulations via the Gillespie algorithm (Gillespie, 1977). For computational efficiency, we implement a form of pseudo-replication in that for each simulation, we sample 100 evenly-spaced sampling centers within the habitat. Each simulation runs for 10 million generations.

Initially, we use these simulations to confirm that a negative binomial PMF provides a good fit to the simulated SFS via the method of moments (Fig. S2-S3). The ouput of each simulation is a vector of sampled values of $P$, which we use to calculate the first two moments of the allele frequency distribution: $\mathbb{E}[P]$ and $\mathbb{E}[P^2]$ (Fig. S4). Having computed these moments, we can then calculate the key parameters of the expected SFS under our model (equivalently to Eq. 44 and Eq. 47:

$$\theta_E = \frac{\mathbb{E}[P]^2}{V[P]}, \tag{70}$$

bioRxiv preprint doi: https://doi.org/10.1101/2024.12.02.626424; this version posted January 29, 2025. The copyright holder for this preprint (which was not certified by peer review) is the author/funder, who has granted bioRxiv a license to display the preprint in perpetuity. It is made available under aCC-BY 4.0 International license.

and

$$\gamma_E = \frac{\mathbb{E}[P]}{V[P]}. \tag{71}$$

We can then use ratios between these terms to compute the $\lambda$ parameter.

### 1.2.2 SLiM simulations

Our second set of simulations implements a previously developed spatial model (Battey et al., 2020) in SLiM (Haller and Messer, 2019). All conditions are the same as in Battey et al. (2020) except that all variants are deleterious with some selection coefficient. For each selection coefficient and sample size of interest, we run 50 replicates of the simulation in a square habitat 75 units wide with a population density of 5 for a genome of length 100 Mbp and mutation rate $1 \times 10^{-10}$ per base pair per generation. For each simulation run, we sample individuals according Gaussian (with varying width) or uniform distributions and obtain the sample SFS. We then average over 100 sampling iterations for each width.

The model from Battey et al. (2020) includes several factors that are not modeled in our theory or branching process simulations. For instance, their model includes non-toroidal boundary conditions, with the probability of individual survival declining near range edges to avoid upward biases in fitness. A particularly notable difference in the two models is the definition of the parent-offspring distribution. In our model, individuals disperse away from their location of origin (which is a single point) at root mean squared distance $\sigma$ per generation. Under the Battey et al. (2020) model, individuals arise as the offspring of two parents, and dispersal occurs from each parent 50% of the time. This results in a constant scalar difference in all spatial parameters between the Battey et al. (2020) model and ours (under our simulation parameters, this scalar works out to 4.08). However, we find that since both $w$ and $\sigma$ parameters in our model are scaled by this factor, it cancels out of $w/\ell_c$ and thus $\lambda$ as well as $\rho\ell_c^2$. As a result, estimates of $\theta_E$ and $\gamma_E$ (and thus, all downstream results) are not changed affected by this difference. We note that time-scales for mutation are the same between models (per-generation).

## 1.3 Extended empirical methods

Here, we provide additional detail on our empirical methods. Scripts are available at: https://github.com/NovembreLab/spatial_rare_alleles. Additional source code is available upon request.

### 1.3.1 Sampling importance resampling algorithm and implementation

A key step in our empirical analysis is to construct samples within the UK Biobank having Gaussian or uniform distribution. Here, we provide detail on the sampling procedure used.

For samples of individuals in geographic (birthplace) space, we first filter the data to individuals passing QC (per UK Biobank metrics Bycroft et al., 2018), born in the UK, with coordinates available, and having Euclidean distance within 0.0001 of the median centroid in PC1-PC2 space. We then calculate binned frequencies of birthplaces in discretized geographic space (20x20 grid).

bioRxiv preprint doi: https://doi.org/10.1101/2024.12.02.626424; this version posted January 29, 2025. The copyright holder for this preprint (which was not certified by peer review) is the author/funder, who has granted bioRxiv a license to display the preprint in perpetuity. It is made available under aCC-BY 4.0 International license.

For uniform samples, we assign weights that are inversely proportional to the frequency of individuals in the bin in which an individual lies. For Gaussian samples, we compute distance per-individual from one of three pre-selected center points (located centrally within a bin) and assign weights according to a Gaussian density with standard deviation $w$, divided by the binned frequency as used in the uniform weight calculation. Centers were chosen to avoid known urban or otherwise high-density areas (to avoid model mis-specification) and such that center locations had sufficient individuals in the nearby region as to avoid extreme re-sampling of individuals. For samples in PCA space, this process is identical except that we use all individuals passing QC, construct the 20x20 grid over PC1-PC2 space, and only use one center point (which corresponds to the bin including the median value). All weights are normalized to sum to one.

Then, using custom scripts, for each set of weights we sample sets $10,000$ individuals *with replacement*. As a result of the weighting scheme used and this sampling step, the birthplace locations/PC1-PC2 coordinates for each sample match either a uniform distribution or a Gaussian distribution with weight $w$, as intended (see, for instance, Fig. S16). We then compute and output the SFS for each sample.

### 1.3.2 Calculation of summary statistics

Having outputted the SFS, we then use it to compute various summary statistics. The number of variant sites, number of singletons, average variant frequency, and average heterozygosity were all calculated using standard formulas. We note that cumulative MAF was calculated following the definition of Wang et al. (2014) using the below formula:

$$\text{MAF}_{cumulative} = 1 - \prod_{i}^{2} n \left(1 - \frac{i}{n}\right)^{x_i} \tag{72}$$

where $x_i$ is the number of alleles at count $i$ in the empirical SFS.

All results shown are averaged over ten sampling replicates each, and values are reported per-kb as appropriate. Additionally, metrics for samples within geographic (birthplace) space are averaged over each of the three sampling centers.

bioRxiv preprint doi: https://doi.org/10.1101/2024.12.02.626424; this version posted January 29, 2025. The copyright holder for this preprint (which was not certified by peer review) is the author/funder, who has granted bioRxiv a license to display the preprint in perpetuity. It is made available under aCC-BY 4.0 International license.

844
# 2 Supplemental Figures

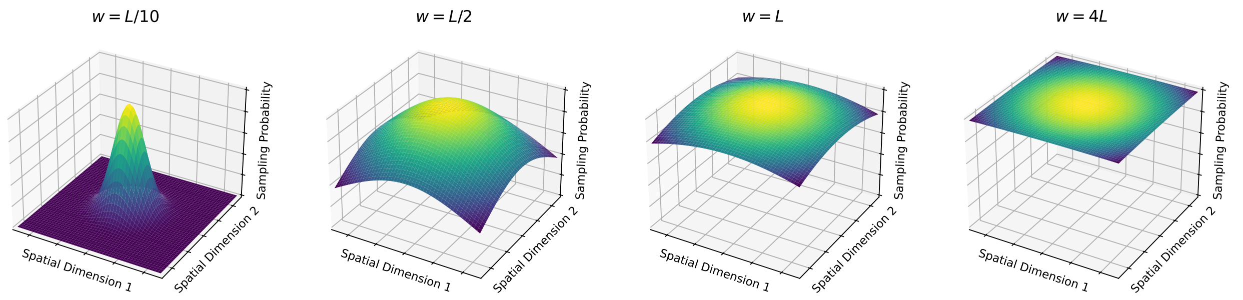


**Figure S1:** Visualization of a Gaussian sampling kernel on a square habitat of size $L \times L$. Values of $w$ are, ranging from "narrow" to "broad".

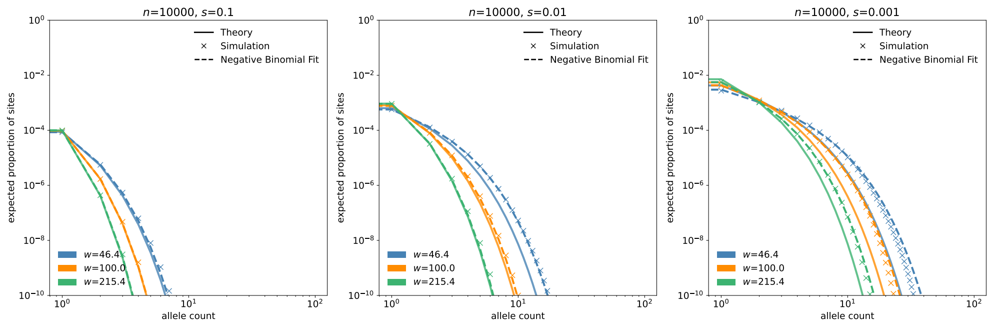


**Figure S2:** Simulated frequency spectra (x's) with sample size $n = 10,000$ for a range of selection coefficients. Dashed lines indicate a negative binomial PMF fit to simulation results via the method of moments. Solid lines indicate theoretical expectation. Other model and simulation parameters include: $\sigma = 10$, $\rho = 20$, $L$=1,000, and $\mu = 1e - 9$.

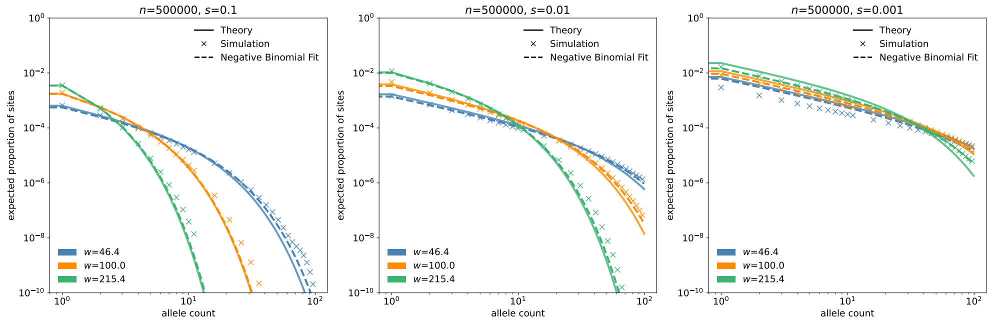


**Figure S3:** Simulated frequency spectra (x's) with sample size $n = 500,000$ for a range of selection coefficients. Dashed lines indicate a negative binomial PMF fit to simulation results via the method of moments. Solid lines indicate theoretical expectation. Other model and simulation parameters include: $\sigma = 10$, $\rho = 20$, $L$=1,000, and $\mu = 1e - 9$.

bioRxiv preprint doi: https://doi.org/10.1101/2024.12.02.626424; this version posted January 29, 2025. The copyright holder for this preprint (which was not certified by peer review) is the author/funder, who has granted bioRxiv a license to display the preprint in perpetuity. It is made available under aCC-BY 4.0 International license.

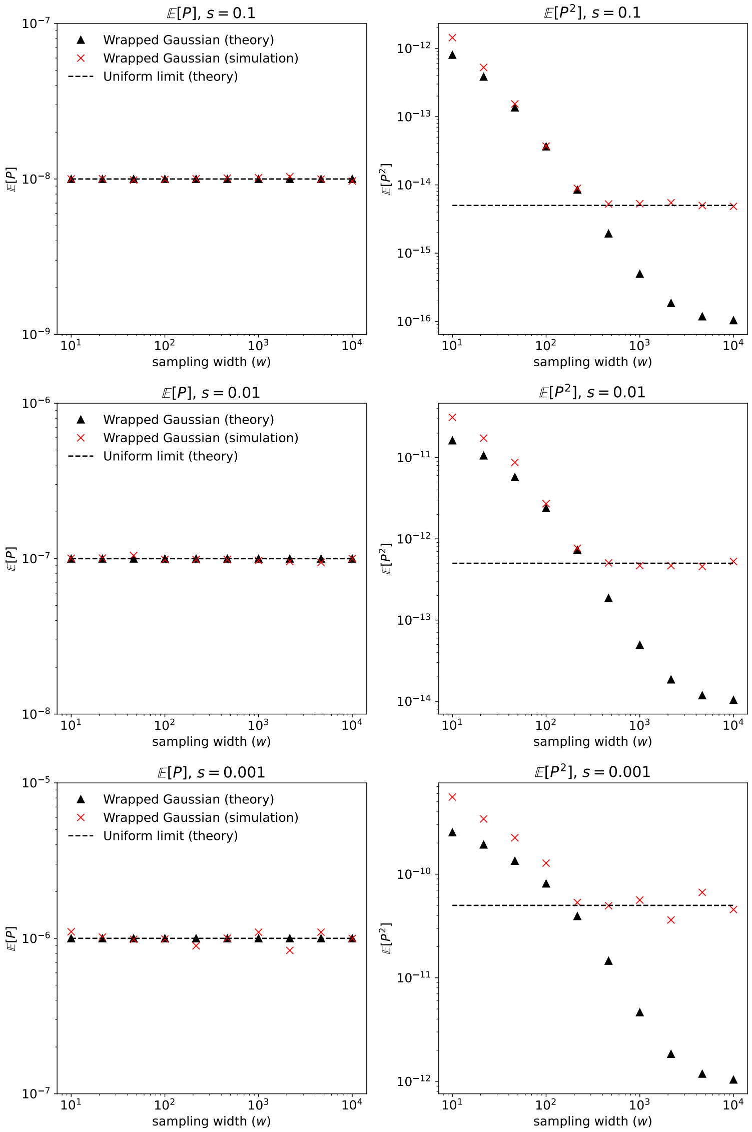


**Figure S4:** Theory and simulation results for the first and second moments of $P$ over a range of sampling widths and selection coefficients. Dashed line shows expectation under uniform sampling, while other markers indicate wrapped Gaussian sampling. Other model and simulation parameters include: $\sigma = 10$, $\rho = 20$, $L$=1,000, and $\mu = 1e - 9$.

bioRxiv preprint doi: https://doi.org/10.1101/2024.12.02.626424; this version posted January 29, 2025. The copyright holder for this preprint (which was not certified by peer review) is the author/funder, who has granted bioRxiv a license to display the preprint in perpetuity. It is made available under aCC-BY 4.0 International license.

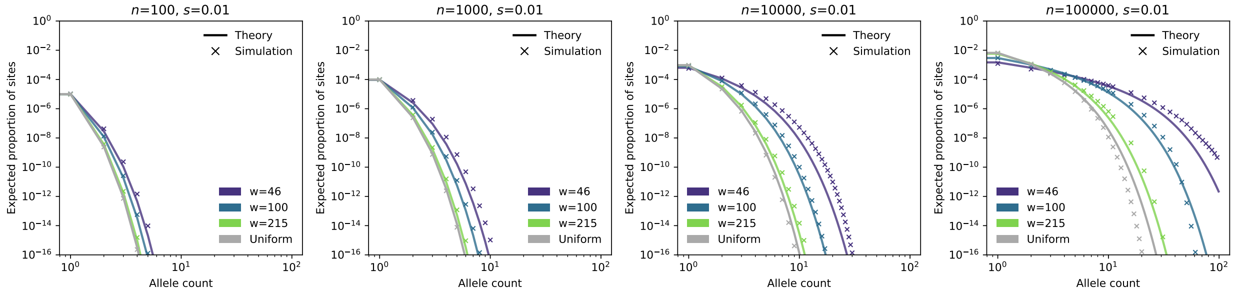


**Figure S5:** Expected site frequency spectrum from theoretical model and branching process simulations for increasing sample size (left to right). Other model and simulation parameters include: $\sigma = 10$, $\rho = 20$, $L$=1,000, and $\mu = 1e - 9$.

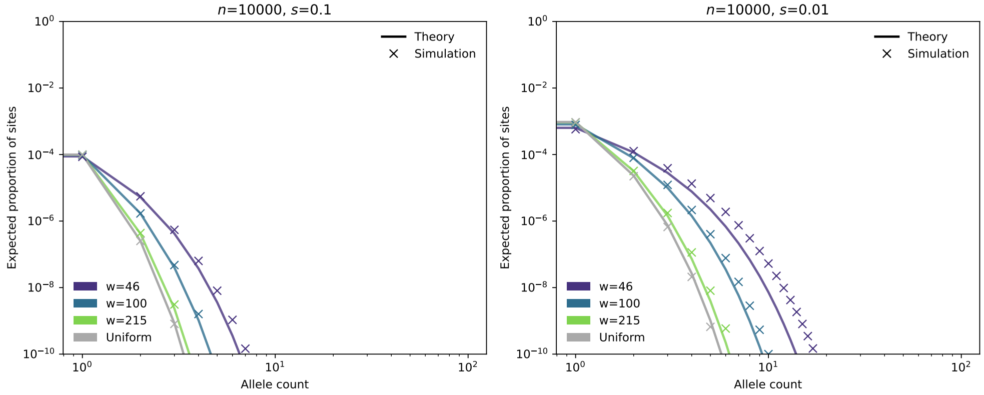


**Figure S6:** Expected site frequency spectrum from theoretical model and branching process simulations for stronger (left) and weaker (right) selection. Other model and simulation parameters include: $\sigma = 10$, $\rho = 20$, $L$=1,000, and $\mu = 1e - 9$.

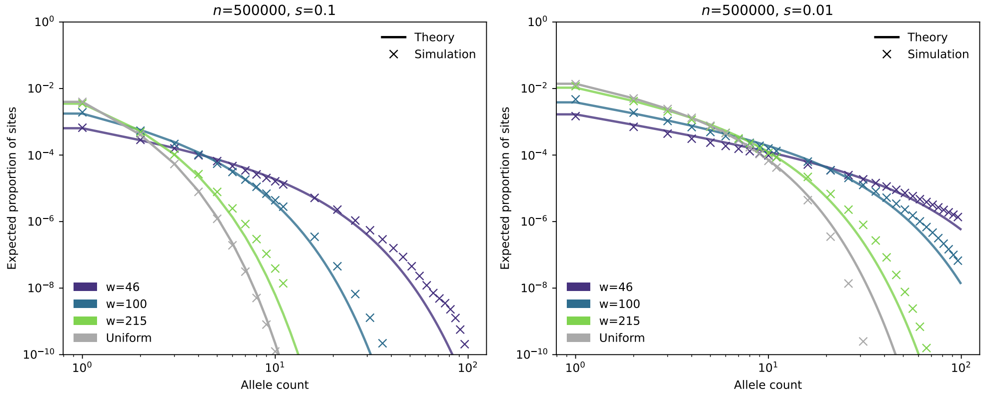


**Figure S7:** Expected site frequency spectrum from theoretical model and branching process simulations for stronger (left) and weaker (right) selection. Other model and simulation parameters include: $\sigma = 10$, $\rho = 20$, $L$=1,000, and $\mu = 1e - 9$.

bioRxiv preprint doi: https://doi.org/10.1101/2024.12.02.626424; this version posted January 29, 2025. The copyright holder for this preprint (which was not certified by peer review) is the author/funder, who has granted bioRxiv a license to display the preprint in perpetuity. It is made available under aCC-BY 4.0 International license.

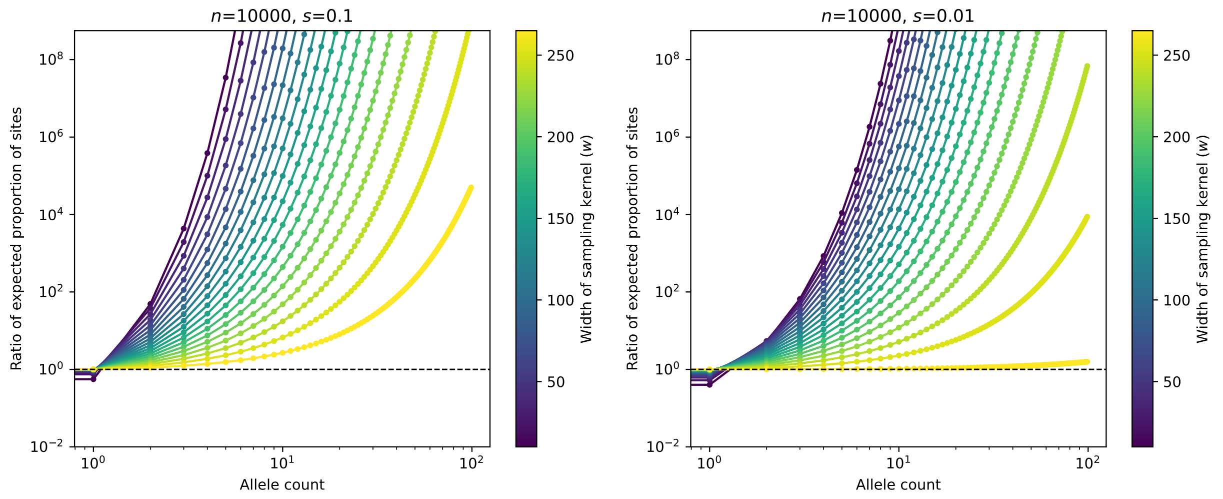


**Figure S8:** Ratio between theoretical SFS values in a sample of width $w$ vs. uniform sampling for stronger (left) and weaker (right) selection. Other model and simulation parameters include: $\sigma = 10$, $\rho = 20$, $L$=1,000, and $\mu = 1e - 9$.

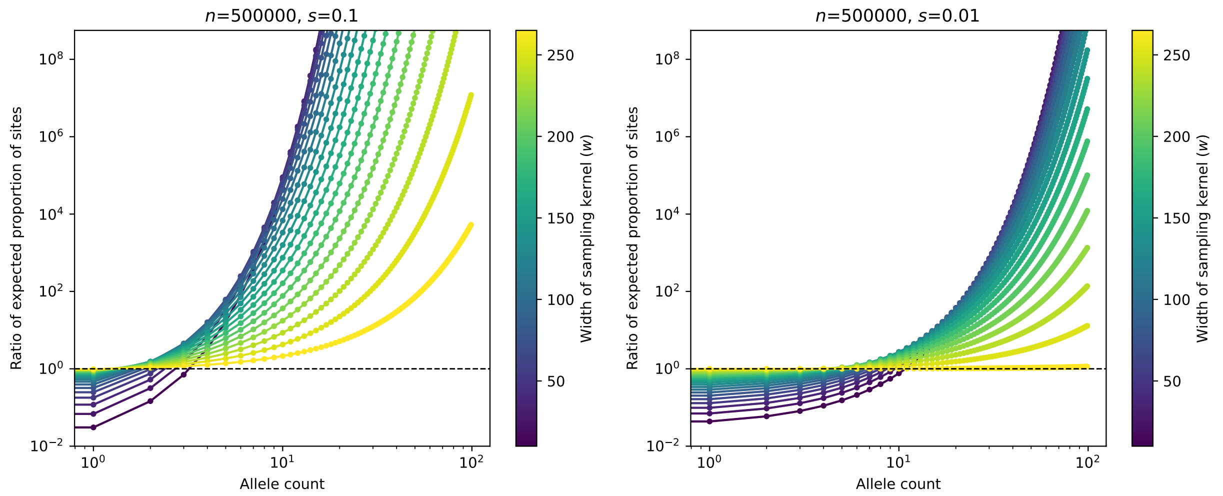


**Figure S9:** Ratio between theoretical SFS values in a sample of width $w$ vs. uniform sampling for stronger (left) and weaker (right) selection. Other model and simulation parameters include: $\sigma = 10$, $\rho = 20$, $L$=1,000, and $\mu = 1e - 9$.

bioRxiv preprint doi: https://doi.org/10.1101/2024.12.02.626424; this version posted January 29, 2025. The copyright holder for this preprint (which was not certified by peer review) is the author/funder, who has granted bioRxiv a license to display the preprint in perpetuity. It is made available under aCC-BY 4.0 International license.

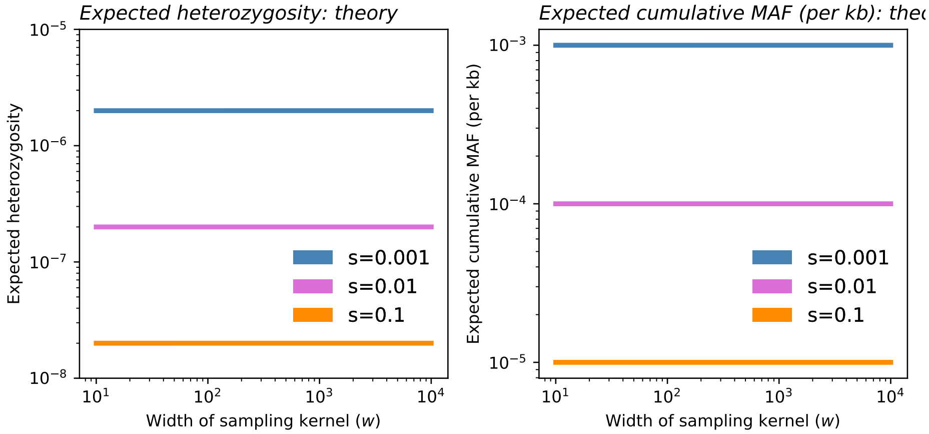


**Figure S10:** Expected heterozygosity and cumulative MAF (per kb) from theory. Inplots shown, $\sigma = 10$, $\rho_N = 20$, and $\mu = 1e - 9$.

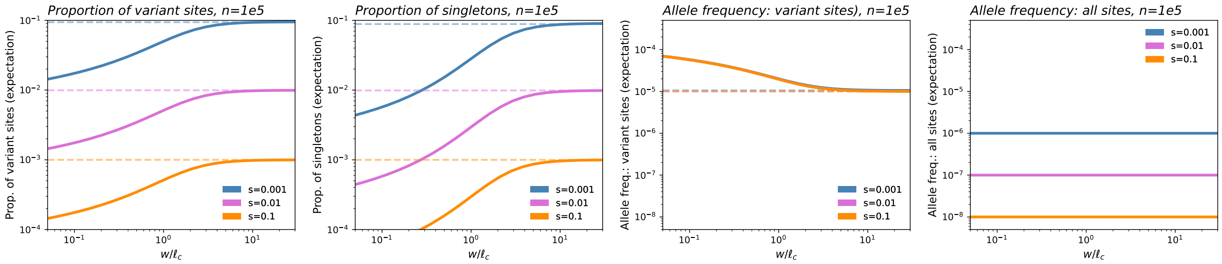


**Figure S11:** Summary statistics, as in Fig. 4, as a function the scaled sampling width $w/\ell_c$. Dashed lines represent theoretical expectation under uniform sampling. Inplots shown, $\sigma = 10$, $\rho_N = 20$, and $\mu = 1e - 9$.

bioRxiv preprint doi: https://doi.org/10.1101/2024.12.02.626424; this version posted January 29, 2025. The copyright holder for this preprint (which was not certified by peer review) is the author/funder, who has granted bioRxiv a license to display the preprint in perpetuity. It is made available under aCC-BY 4.0 International license.

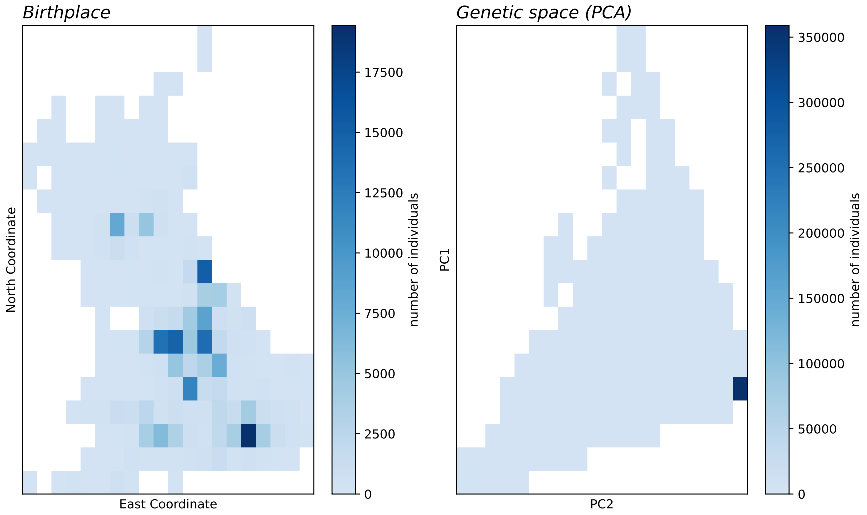


**Figure S12:** Counts of individuals in the UK Biobank included in empirical analyses over discretized geographic (left) and genetic (right) space. Each grid has dimensions 20x20 with equal-sized bins.

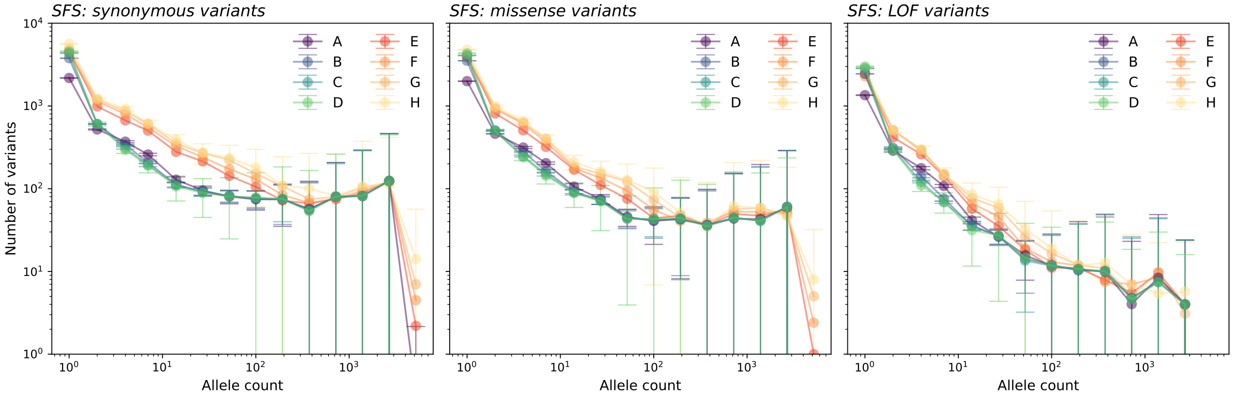


**Figure S13:** Site frequency spectra for sampling distributions as depicted in Fig. 3 across three variant classes.

bioRxiv preprint doi: https://doi.org/10.1101/2024.12.02.626424; this version posted January 29, 2025. The copyright holder for this preprint (which was not certified by peer review) is the author/funder, who has granted bioRxiv a license to display the preprint in perpetuity. It is made available under aCC-BY 4.0 International license.

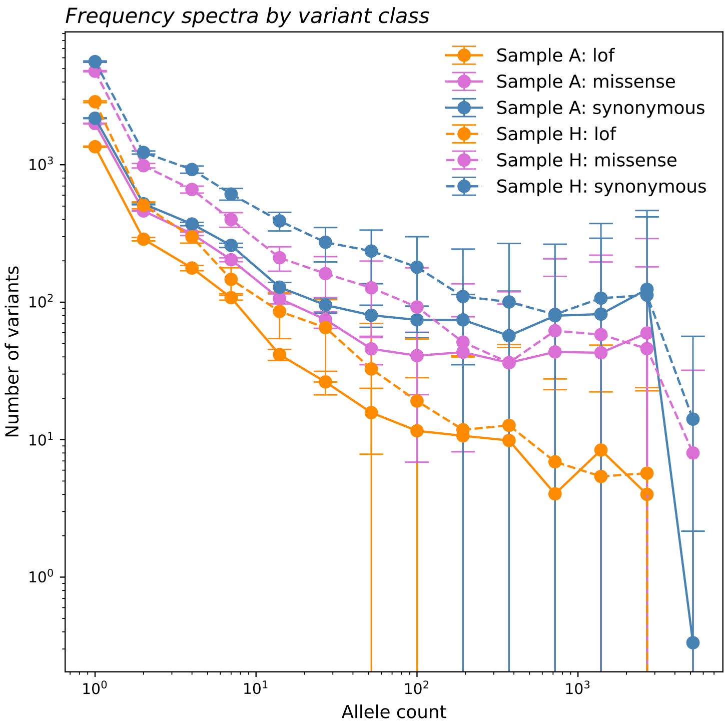


**Figure S14:** Site frequency spectra for narrowest (**A**) and broadest (**H**) sampling distributions across all three variant classes.

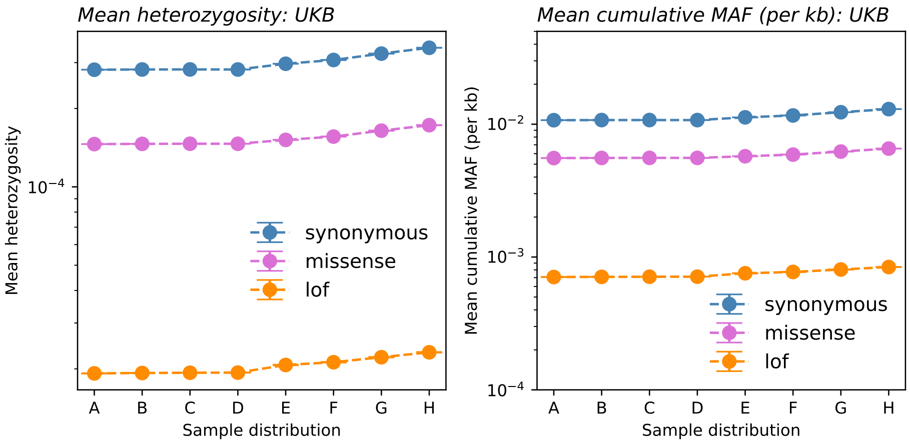


**Figure S15:** Mean heterozygosity (left) and cumulative MAF (per kb; right) as calculated from UKB re-samples.

bioRxiv preprint doi: https://doi.org/10.1101/2024.12.02.626424; this version posted January 29, 2025. The copyright holder for this preprint (which was not certified by peer review) is the author/funder, who has granted bioRxiv a license to display the preprint in perpetuity. It is made available under aCC-BY 4.0 International license.

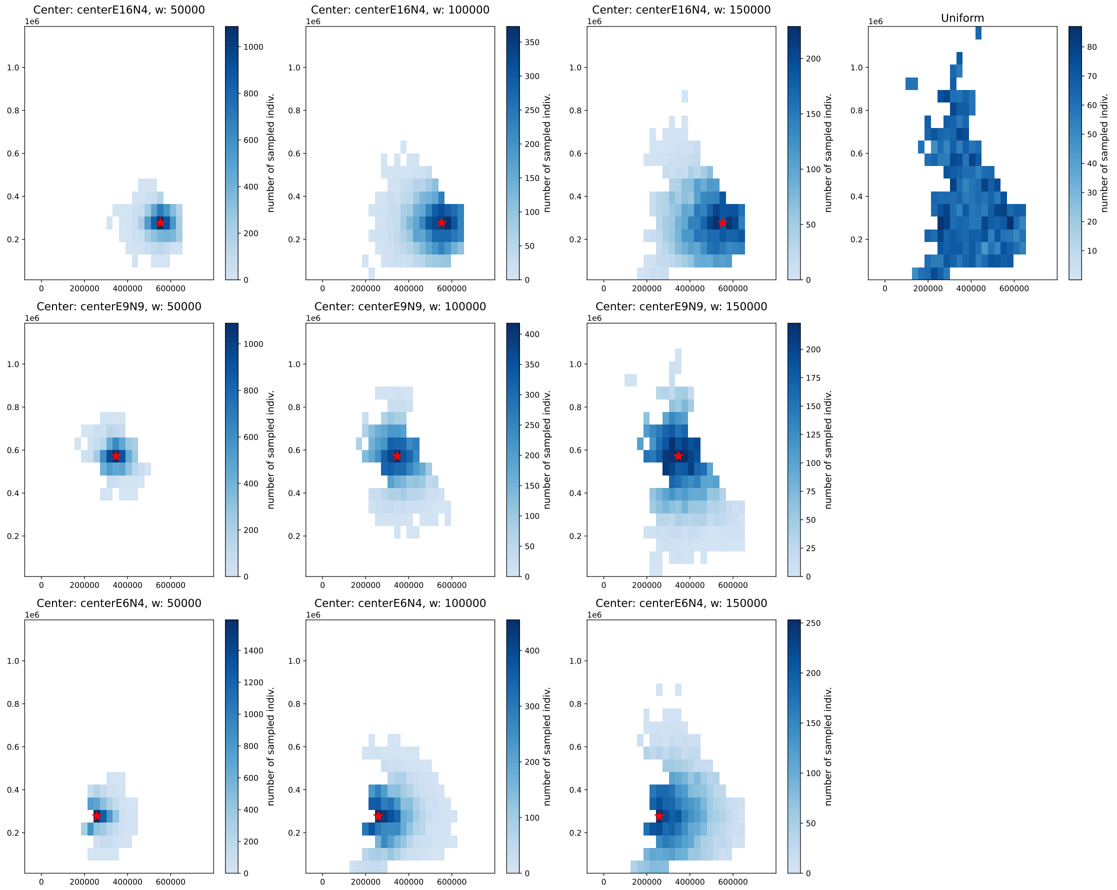


**Figure S16:** Visualization of sampling distributions in geographic space across three center points and values of $w$ (as well as the uniform distribution).
